# PINK1 contained in huMSC-derived exosomes prevents cardiomyocyte mitochondrial calcium overload in sepsis via recovery of mitochondrial Ca^2+^ efflux

**DOI:** 10.1186/s13287-021-02325-6

**Published:** 2021-05-06

**Authors:** Qin Zhou, Min Xie, Jing Zhu, Qin Yi, Bin Tan, Yasha Li, Liang Ye, Xinyuan Zhang, Ying Zhang, Jie Tian, Hao Xu

**Affiliations:** 1 Department of Pediatric Research Institute, Ministry of Education Key Laboratory of Child Development and Disorders, National Clinical Research Center for Child Health and Disorders (Chongqing), China International Science and Technology Cooperation base of Child development and Critical Disorders, Children’s Hospital of Chongqing Medical University, Chongqing, People’s Republic of China; 2 Chongqing Key Laboratory of Pediatrics, Chongqing, People’s Republic of China; 3 Department of Cardiovascular (Internal Medicine), Ministry of Education Key Laboratory of Child Development and Disorders, National Clinical Research Center for Child Health and Disorders (Chongqing), China International Science and Technology Cooperation base of Child development and Critical Disorders, Children’s Hospital of Chongqing Medical University, Chongqing, People’s Republic of China; 4 Department of Clinical Laboratory, Ministry of Education Key Laboratory of Child Development and Disorders, National Clinical Research Center for Child Health and Disorders (Chongqing), China International Science and Technology Cooperation base of Child development and Critical Disorders, Children’s Hospital of Chongqing Medical University, Box 136, No. 3 Zhongshan RD, Yuzhong district, Chongqing, 400014 People’s Republic of China

**Keywords:** Sepsis, Cardiac dysfunction, PINK1, Calcium overload, Mitochondrial Ca^2+^ efflux

## Abstract

**Background:**

Sepsis is a systemic inflammatory response to a local severe infection that may lead to multiple organ failure and death. Previous studies have shown that 40–50% of patients with sepsis have diverse myocardial injuries and 70 to 90% mortality rates compared to 20% mortality in patients with sepsis without myocardial injury. Therefore, uncovering the mechanism of sepsis-induced myocardial injury and finding a target-based treatment are immensely important.

**Objective:**

The present study elucidated the mechanism of sepsis-induced myocardial injury and examined the value of human umbilical cord mesenchymal stem cells (huMSCs) for protecting cardiac function in sepsis.

**Methods:**

We used cecal ligation and puncture (CLP) to induce sepsis in mice and detect myocardial injury and cardiac function using serological markers and echocardiography. Cardiomyocyte apoptosis and heart tissue ultrastructure were detected using TdT-mediated dUTP Nick-End Labeling (TUNEL) and transmission electron microscopy (TEM), respectively. Fura-2 AM was used to monitor Ca^2+^ uptake and efflux in mitochondria. FQ-PCR and Western blotting detected expression of mitochondrial Ca^2+^ distribution regulators and PTEN-induced putative kinase 1 (PINK1). JC-1 was used to detect the mitochondrial membrane potential (Δψm) of cardiomyocytes.

**Results:**

We found that expression of PINK1 decreased in mouse hearts during sepsis, which caused cardiomyocyte mitochondrial Ca^2+^ efflux disorder, mitochondrial calcium overload, and cardiomyocyte injury. In contrast, we found that exosomes isolated from huMSCs (huMSC-exo) carried Pink1 mRNA, which could be transferred to recipient cardiomyocytes to increase PINK1 expression. The reduction in cardiomyocyte mitochondrial calcium efflux was reversed, and cardiomyocytes recovered from injury. We confirmed the effect of the PINK1-PKA-NCLX axis on mitochondrial calcium homeostasis in cardiomyocytes during sepsis.

**Conclusion:**

The PINK1-PKA-NCLX axis plays an important role in mitochondrial calcium efflux in cardiomyocytes. Therefore, PINK1 may be a therapeutic target to protect cardiomyocyte mitochondria, and the application of huMSC-exo is a promising strategy against sepsis-induced heart dysfunction.

**Supplementary Information:**

The online version contains supplementary material available at 10.1186/s13287-021-02325-6.

## Introduction

Sepsis is a systemic inflammatory response to a local severe infection that may lead to multiple organ failure and death, especially in patients with cardiac dysfunction, which increases mortality to 70–90% compared to patients without cardiac dysfunction [1]. Sepsis-induced cardiac injury or dysfunction subsequently contributes to cardiovascular collapse, which results in poor perfusion of blood into multiple tissues [2]. Therefore, examining the mechanism of sepsis-induced cardiac injury and finding a way to protect the heart from injury during sepsis would provide beneficial effects on mortality in this complex disease.

The mechanisms underlying cardiac dysfunction in sepsis are not completely understood but include the following main mechanisms: (a) increased release of pro-inflammatory cytokines [3], (b) apoptotic myocardial cell death via activation of extrinsic and intrinsic pathways, (c) metabolic disorders and inducible nitric oxide synthase (NOS)-dependent action [4], (d) cardiac-depressant action of lysozyme c primarily originating from disintegrating neutrophils and monocytes [5], and (e) mitochondrial dysfunction [6, 7]. Previous studies observed mitochondrial structural abnormalities at the early stage of sepsis, and mitochondrial respiration function was reduced significantly and caused a reduction in ATP production, which led to myocardial contractility dysfunction [8, 9]. However, there are few studies on the mechanism of cardiomyocyte mitochondrial dysfunction during sepsis.

In the early stage of sepsis, an abundance of Ca^2+^ flow into the cytoplasm of cardiomyocytes from the extracellular space, and the mitochondrial uptake creates an overload of Ca^2+^ in the matrix, which ultimately induces mitochondrial calcium overload. Mitochondrial injury induced by calcium overload is an important mechanism of heart dysfunction in sepsis. The obstruction of mitochondrial Ca^2+^ (_m_Ca^2+^) efflux mediated by the mitochondrial Na^+^/Ca^2+^ exchanger (NCLX) is an important factor in mitochondrial calcium overload [10]. The activity of NCLX is regulated by several proteins and kinases, such as stomatin-like protein 2 (SLP-2) and protein kinase C (PKC) [11, 12]. In a cell model of Parkinson’s disease related to mutations in PTEN-induced putative kinase 1 (PINK1), the failure of mitochondrial Ca^2+^ efflux in neurons deficient in PINK1 was linked to the impaired activity of NCLX, which resulted in mitochondrial Ca^2+^ overload and suggested that PINK1 regulated the activity of NCLX [13, 14]. Therefore, whether disorders of PINK1 and NCLX underlie cardiomyocyte mitochondrial dysfunction and whether reducing mitochondrial Ca^2+^ overload would rescue the cardiac injury are worthy of investigation.

Mesenchymal stem cells (MSCs) effectively reduce mortality and improve myocardial function in animal models of sepsis [15–17]. However, MSCs primarily migrate to the lung and liver after systemic infusion in these models, and few cells are detected in cardiac tissue [18, 19]. Therefore, MSC-induced cardiac benefits during sepsis may not be related to local actions but to paracrine effects from a distance [20]. The beneficial effects of MSCs are mediated by paracrine factors, such as cytokines, growth factors, and extracellular vesicles. Exosomes are extracellular vesicles that mediate local and systemic cell-to-cell communication. These vesicles are 30–100-nm-sized membrane vesicles that transfer specific sets of functional RNAs (miRNAs, mRNAs, and lncRNAs) and proteins to recipient cells [21]. Several studies have shown that MSC-derived exosomes (MSC-exos) improved recovery in animal models of liver fibrosis, kidney failure, and myocardial ischemia/reperfusion injury [20, 22, 23]. Xiaohong Wang et al. reported that MSC-exos also contributed to cardioprotective effects in sepsis [20], but how MSC-exos exert cardioprotective effects during sepsis was not elucidated.

Based on the current theoretical framework, we hypothesized that sepsis-induced cardiac dysfunction may be caused by cardiomyocyte mitochondrial calcium overload, and MSC-exos would rescue injured mitochondria by reducing calcium overload. The present study examined the mechanism of cardiomyocyte mitochondrial calcium overload during sepsis and investigated whether MSC-exos protected heart function during sepsis by reducing mitochondrial damage and whether MSC-exos repaired mitochondrial damage via PINK1 and NCLX.

## Methods

### Animals and sepsis model

C57BL/6 mice aged 6–8 weeks were obtained from and raised at Chongqing Medical University. Mice were randomly assigned to three groups: sham, cecal ligation and puncture (CLP) and CLP + exo. Sixteen mice were used for each group: 6 mice were used to detect serum biomarkers and ACM isolation, 3 of these mice underwent echocardiography before serum collection, and 10 mice were used to measure survival rate. CLP was performed to establish a model of sepsis [24]. Briefly, the animals were anesthetized with pentobarbital (1%, 50 mg/kg body weight i.p.), and small scissors were used to make the incision and gain entry into the peritoneal cavity. The cecum was located, exteriorized, ligated, and punctured using a 26-gauge needle at the designated position to induce mid-grade sepsis. The cecum was relocated into the abdominal cavity, and the incision was closed. The sham group was exposed to the same surgery, but the cecum was not ligated or punctured. The CLP + exo group were treated with exosomes isolated from human MSCs (huMSCs) after CLP. Serum biomarkers and echocardiography were used to evaluate sepsis-induced heart injury [9].

### Echocardiography

Mice in each group were administered an anesthetic at the corresponding time point after modeling. The chest skin was depilated, and the mice were placed on a plate to maintain anesthesia and sedation. Transthoracic echocardiography was performed using a high-frequency, high-resolution digital imaging system (Vevo 2100 Imaging System, Fujifilm Visualsonics, Canada) with a transducer probe (VisualSonics 550D, Fujifilm Visualsonics, Canada). A thick gel was applied to the chest, the probe was placed on the left edge of the sternum, and two-dimensional B-mode ultrasound was used to display the standard left ventricular short-axis view. The probe was moved to the level of the papillary muscles, and M-mode ultrasound was used to record the left ventricular motion curve. A single trained individual performed all echocardiograms.

### Serological markers of myocardial injury detection

At least 500 μL of whole blood per mouse was collected in heparin, and the plasma was collected after centrifugation at 8000 rpm for 10 min. The levels of α-hydroxybutyrate dehydrogenase (HBDH) and creatine kinase (CK) in plasma were measured using an automatic biochemical analyzer (C701, Roche, Switzerland). Cardiac troponin-I (cTnI) was quantified using ELISA (E-EL-M1203c, Elabscience, China).

### Cell culture and treatments

The huMSCs used in our study were obtained from Chongqing Stem Cell Therapy Engineering and Technology Center and cultured in DMEM/F12 supplemented with 10% fetal bovine serum (FBS; Millipore, USA). Adult mouse cardiomyocytes (ACMs) were isolated from adult mouse hearts and cultured in dishes or plates precoated with mouse laminin [25]. Human ventricular myocyte AC16 cells (Otwo Biotech, ShenZhen, China) were cultured in DMEM supplemented with 10% fetal bovine serum and treated with lipopolysaccharide (LPS, 5 μg/ml, Solarbio, L8880, China) to create an in vitro sepsis model. The AC16 cells were treated with forskolin (20 μM, 12 h, Selleck, S2449, USA) or the protein kinase A (PKA) inhibitor H89 (5 μM, 12 h, Selleck, S1582, USA).

### Transient transfection with siRNA

The huMSCs were transfected with siRNA according to the manufacturer’s instructions. Transfection of siRNA into huMSCs was performed using RNAiMax reagent (Thermo Fisher Scientific, USA) at a final concentration of 100 nM. Cellular RNA, protein, and culture medium supernatant collection were performed 48 h after transfection. The siRNA sequence for *Pink1* was 5′-GGCTGGTGATCGCAGATTT-3′.

### Isolation and characterization of exosomes

The huMSCs were maintained under normal conditions until they reached 70 to 80% confluency. Twenty-four hours before the collection of exosomes from huMSCs and huMSCs transfected with *Pink1* siRNA, the medium was replaced with medium containing 10% exosome-depleted FBS. Supernatants were collected and centrifuged successively at 300×*g* for 10 min, 2000×*g* for 10 min, and 10,000×*g* for 30 min, and the supernatants were transferred to a new tube at each step. The final supernatant was ultracentrifuged at 100,000×*g* for 70 min, and the pellet was washed in PBS and ultracentrifuged at 100,000×*g* for another 70 min [26]. The pellet was resuspended in PBS and quantified using the BCA assay. The mice were treated with 2 μg/g exosomes at 0 h and 6 h after CLP respectively and cocultured with cells at 2 μg/ml. The quality of the exosomes was confirmed using transmission electron microscopy (TEM), particle size assessment (ZEN3600, Malvern, UK), and exosome markers. To monitor exosome trafficking, exosomes were labeled with PKH26 fluorescent dye using the PKH26 fluorescent cell linker kit (Sigma-Aldrich, MKCJ1898).

### Fluorescence quantitative PCR

Total RNA was isolated from rat hearts using TRIzol (Takara, Japan) according to the manufacturer’s instructions. cDNA was generated using 1 μg RNA with a PrimeScriptTM RT Reagent Kit (Takara, Japan). FQ-PCR was performed using TB Green (Takara, Japan) with a CFX-96 (BIO-RAD, USA). We evaluated samples for mRNA expression of *Mcu*, *Micu1*, *Nclx*, and *Pink1*. All experimental samples were analyzed in triplicate and averaged. To calculate the fold change in mRNA expression, the 2^−ΔΔCt^ method was used.

### Western blotting

Proteins were isolated from rat hearts using lysis buffer (KeyGEN BioTECH, China) with a protease and phosphatase inhibitor cocktail solution (KeyGEN BioTECH, China). Thirty micrograms of protein was loaded on a 10% PAGE Gel Fast Preparation Kit (PG112, Epizyme Biotech, China). Proteins were separated and transferred to PVDF membranes (Millipore, USA). Membranes were incubated in TBST skim milk blocking buffer for 1 h at room temperature (RT) followed by primary antibodies overnight at 4 °C. The following primary antibodies were used: CD81 (sc-166,029, Santa Cruz, USA), Alix (sc-53,540, Santa Cruz, USA), MCU (26312-1-AP, Proteintech, USA), MICU1 (ab224161, Abcam, England), NCLX (ab83551, Abcam, England), PINK1 (23274-1-AP, Proteintech, USA), and GAPDH (AF7021, Affinity Biosciences, USA). Membranes were washed with TBST three times, incubated with secondary antibodies (701051, Zen Bioscience, China; ZB-2301, ZSGB-BIO, China) for 1 h at RT and washed with TBST three times. Membranes were probed using a chemiluminescence kit (Millipore, USA) and an Image system (Bio-Rad, USA).

### Protein kinase A (PKA) activity assay

PKA activity was examined using PKA Kinase Assay Kits, Type I (Immunechem, ICP0216) according to the manufacturer’s protocol. Briefly, the supernatant (50 μl/well) of samples was added to the substrate plate containing kinase assay dilution buffer. ATP (10 μl/well) was added to initiate the kinase reaction at 30 °C for 90 min. The reaction solution was removed and anti-p-substrate antibodies (40 μl/well) were incubated for 60 min at RT. Goat anti-rabbit IgG HRP was used as a secondary antibody, and TMB solution was used to indicate reaction activity. Relative kinase activity was detected at OD450 and normalized to the protein concentration, which was measured using the BCA method.

### TdT-mediated dUTP Nick-End Labeling (TUNEL)

The tissue was fixed in 4% paraformaldehyde for at least 24 h, embedded in paraffin and sectioned into 4-μm slices. Apoptosis was assessed using a TUNEL kit (KGA702-1, KeyGEN BioTECH, China) according to the manufacturer’s instructions. Images were acquired on a Nikon microscope (90i, Japan).

### TEM

Heart tissue was fixed with a 4% glutaraldehyde solution for 2 h and postfixed with 1% osmium tetroxide for 2 h at 4 °C. The fixed tissue was rinsed with distilled water, dehydrated in an ethanol and methanol gradient, and embedded in epoxy resin. The samples were sectioned and contrast-stained for imaging. TEM images were acquired at random locations throughout the samples. Micrographs were taken with a transmission electron microscope (H-7500).

### Evaluation of _m_Ca^2+^ uptake and efflux

ACMs or AC16 (300000) cells were transferred to an intracellular-like medium containing 120 mM KCl, 10 mM NaCl, 1 mM KH_2_PO_4_, 20 mM HEPES-Tris, 3 mM thapsigargin, 80 μg ml^−1^ digitonin, protease inhibitors, and 10 μM succinate at pH 7.2. Fura-2 AM (1 μM) was added to monitor extramitochondrial Ca^2+^. Fluorescence signals were monitored at excitations of 340 and 380 nm and an emission of 510 nm for Fura-2 to calculate ratiometric changes. At 60 s, a 20-μM Ca^2+^ bolus was added. Clearance of extramitochondrial Ca^2+^ was representative of _m_Ca^2+^ uptake. At 260 s, 1 μM Ru360 (MCU inhibitor) was added to inhibit uptake and allow for quantification of _m_Ca^2+^ efflux. At 360 s, 10 μM CGP-37157 (NCLX inhibitor) was added to block _m_Ca^2+^ efflux. FCCP was added at the completion of the experiment. All experiments were performed at 37 °C and recorded on a Cytation 5 (Biotek, USA). The details were reported previously [10].

### Mitochondrial membrane potential assay

The mitochondrial membrane potential was measured using a mitochondrial membrane potential assay kit (JC-1, Beyotime Biotech, China) according to the manufacturer’s instructions. Cells were incubated with medium mixed with JC-1 working fluid in a 37 °C incubator for 20 min. The cells were washed with JC-1 buffer three times and replaced with fresh medium. Fluorescence was captured using confocal microscopy (A1R, Nikon, Japan). The ratio of red and green fluorescence was analyzed by an NIS-Elements system (Nikon, Japan). A high ratio indicated a high mitochondrial membrane potential.

### Statistical analysis

Each experiment was repeated at least three times. All data are expressed as the means ± SD, and the statistical evaluations were performed using *t* tests with independent samples. One-way analysis of variance (ANOVA) with Tukey’s multiple comparisons test was used between groups. SPSS 17.0 software (SPSS Inc., Armonk, NY, USA) was used for statistical analyses. For all analyses, a value of *p* < 0.05 was considered significant.

## Results

### Sepsis-induced heart injury and huMSCs-exo showed cardioprotective effects

**Fig. 1:**
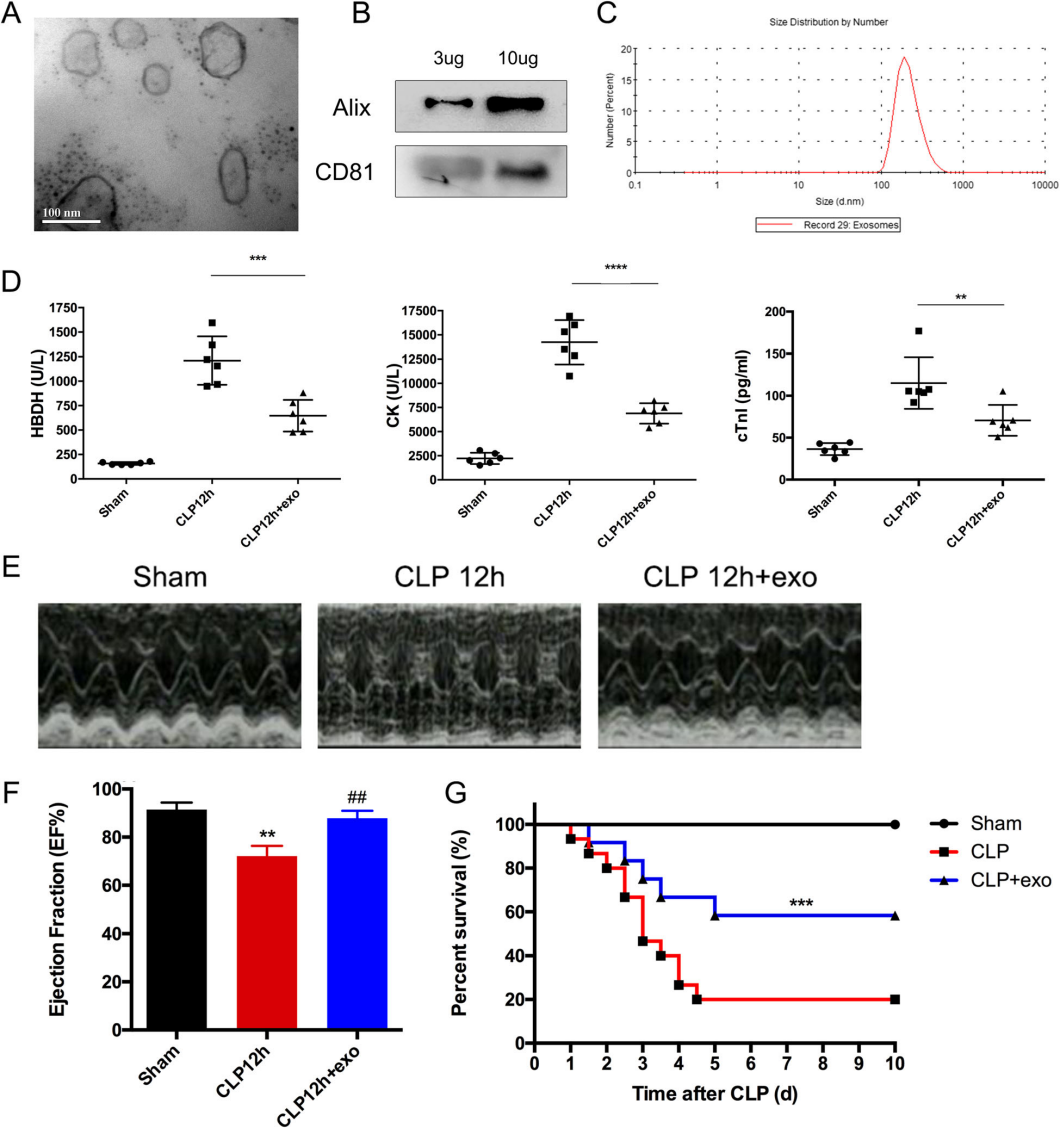
HuMSC-exos showed cardioprotective effects in septic mice. **a**–**c** Characterizations of exosomes derived from huMSCs. **a** Electron micrographs of huMSCs exosomes. **b** The size of huMSC exosomes measured using a particle size analyzer. **c** Exosome markers detected by Western blot. **d** Detection of myocardial injury serological markers HBDH, CK, and cTnI; *p < 0.05, **p < 0.01, ****p < 0.0001 when compared between mice 12 h after CLP and the huMSC-exo treatment group. **e** Representative echocardiography of the sham, CLP 12 h, and CLP 12 h + exo groups. **f** Measurement of ejection fraction; **p < 0.01 vs. sham group, ^##^*p* < 0.01 vs. CLP 12 h group. **g** Survival in mice following huMSC-exo treatment after CLP. Comparisons between groups were performed using Kaplan-Meier analysis followed by log-rank tests; ***p < 0.001 vs. CLP group

**Fig. 2:**
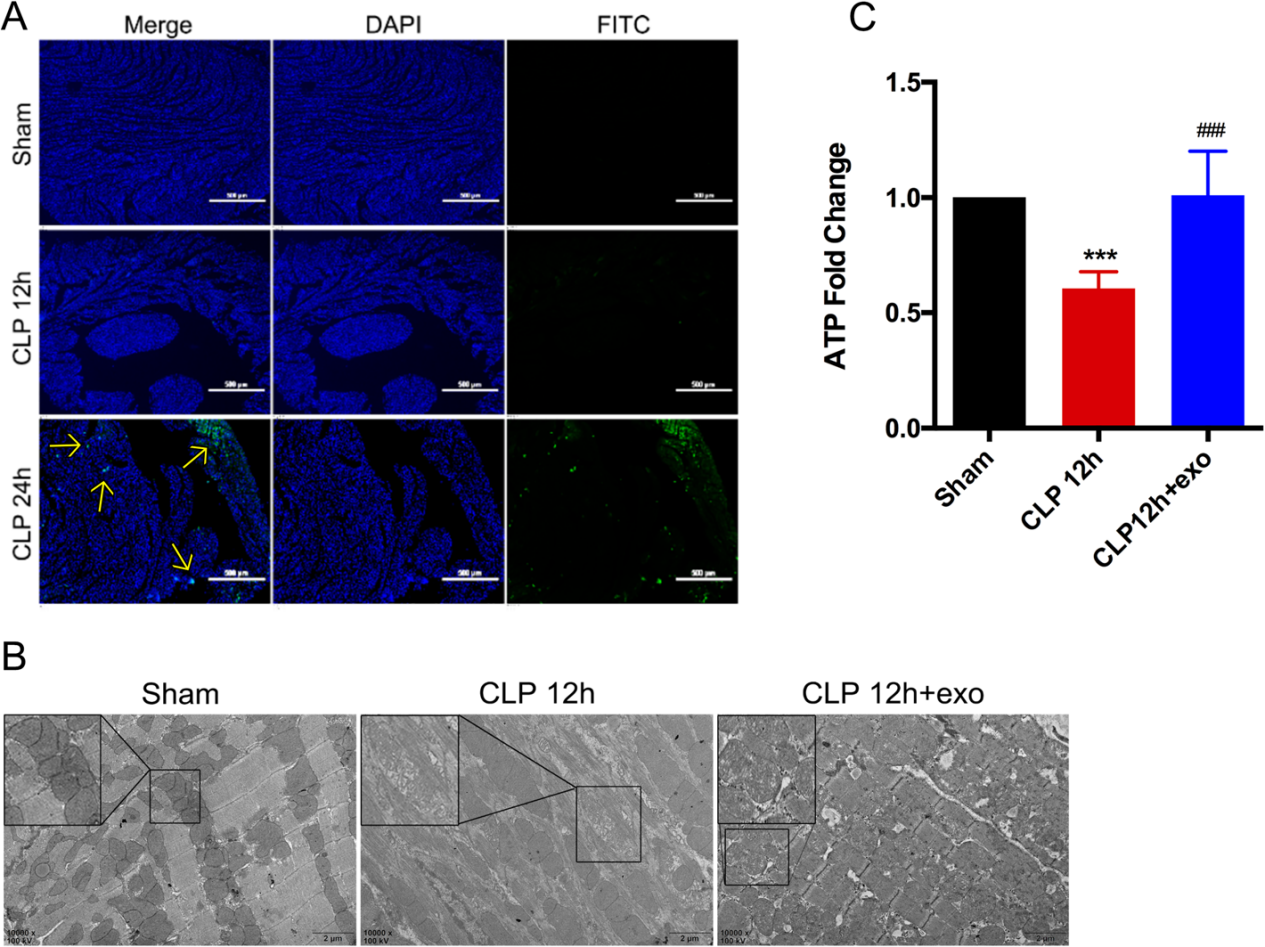
Sepsis-induced heart dysfunction is caused by mitochondrial injury, which is reversed by huMSCs-exo. **a** Representative TUNEL staining for the detection of apoptotic cardiomyocytes (green dots indicated by arrows); nuclei were stained with DAPI (blue). **b** After CLP for 12 h and treatment with huMSCs-exo, the mitochondrial architecture was observed under transmission electron microscopy (TEM) and imaged at a × 10,000 magnification (scale bars, 2 μm); the box in the image is a local magnification. **c** The cardiomyocyte ATP content in mice 12 h after the CLP and treatment with huMSC-exo was measured using a bioluminescent assay system; ***p < 0.001 vs. sham group, ###*p* < 0.001 vs. CLP 12 h group

HuMSCs-exo were isolated and purified via differential ultracentrifugation and assessed using TEM, exosome markers, and particle size (Fig. 1a–c, FigS1 A). We used CLP to investigate the cardioprotective effects of huMSCs-exo in septic mice. A 2-μg/g concentration of huMSCs-exo was intraperitoneally injected 0 h and 6 h after CLP, followed by testing at 12 h after CLP. We tracked the labeled exosomes by Confocal Microscopy (FigS2) and confirmed the cardiomyocyte uptake the exosomes at 12 h after CLP.

We found that the serum markers of cardiomyocyte injury (HBDH, CK and cTnI) increased significantly after CLP (Fig. 1d, HBDH: CLP 12 h group 1210 ± 246.2 versus sham group 158.2 ± 14.41, *p* < 0.0001; CK: CLP 12 h group 14,238 ± 2293 versus sham group 2229 ± 582.7, *p* < 0.0001; cTnI: CLP 12 h group 115.1 ± 30.8 versus sham group 36.35 ± 7.167, *p* < 0.0001), which indicated that sepsis induced cardiomyocyte injury in the first 12 h. In contrast, these markers decreased markedly in the huMSCs-exo treatment group (Fig. 1d, HBDH: CLP 12 h + exo group 647 ± 162.2 versus CLP 12 h group 1210 ± 246.2, *p* = 0.0001; CK: CLP 12 h + exo group 6884 ± 1051 versus CLP 12 h group 14,238 ± 2293, *p* < 0.0001; cTnI: CLP 12 h + exo group 70.66 ± 18.4 versus CLP 12 h group 115.1 ± 30.8, *p* = 0.0063), which indicated that treatment with huMSCs-exo effectively mitigated sepsis-induced cardiomyocyte injury. We detect heart function measured by echocardiography during sepsis (Fig. 1e, f, Table S1). The results showed that EF decreased notably at 12 h after CLP, and huMSCs-exo significantly improved EF compared to the CLP group (Fig. 1e, f, CLP 12 h group 72.11 ± 4.231% versus sham group 91.49 ± 2.925%, *p* = 0.0012, and CLP 12 h + exo group 87.84 ± 3.127%, *p* = 0.0035). These results indicated that huMSCs-exo prevented sepsis-induced heart function disorder. CLP mouse survival also increased significantly after huMSC-exo treatment compared to the untreated group (Fig. 1g).

### Cardiomyocyte mitochondrial damage caused by sepsis can be reversed by huMSCs-exo

To investigate the extent of cardiomyocyte injury in early sepsis, we used TUNEL to measure cardiomyocyte apoptosis. We found that serum markers of cardiomyocyte injury increased significantly, but there was almost no apoptosis in cardiomyocytes 12 h after CLP, and some cardiomyocytes did not undergo apoptosis until 24 h after CLP (Fig. 2a). This result suggests that cardiomyocytes were in the early stage of apoptosis 12 h after sepsis, and some factors increased cardiomyocyte membrane permeability and resulted in cardiac-specific protein release.

To reveal the reason for increased injury markers and identify changes in cardiomyocytes during the first 12 h of sepsis, we observed cardiomyocyte ultrastructure using TEM. We observed a disorderly myocardial myofibril arrangement, and the band area was blurred, broken, or dissolved 12 h after CLP despite the absence of cardiomyocyte apoptosis. We also observed that cardiomyocyte mitochondrial swelling, cristae disorganization, and cristae number decreased, which indicated that cardiomyocyte mitochondria were seriously injured during the first 12 h of sepsis (Fig. 2b). One of the main characteristics of mitochondrial dysfunction is reduced ATP production, which was consistent with our results, and we found that ATP production was significantly reduced 12 h after CLP (Fig. 2c, CLP 12 h group 0.6058 ± 0.07267 versus sham group 1 (100%), *p* = 0.0005).

We hypothesized that the cardioprotective effects of huMSC-exo were mediated via mitochondria protection. Therefore, we examined cardiomyocyte ultrastructure using TEM after treatment with huMSC-exo and found that myocardial myofibrils were arranged in order with no broken or dissolved myofibrils. The mitochondria morphology was similar to the sham group, with only a few mitochondria showing a decreased number of cristae (Fig. 2b). This observation indicates that huMSC-exo effectively prevented cardiomyocyte mitochondrial injury in the first 12 h of sepsis. The ATP production of cardiomyocytes in the treatment group 12 h after CLP also increased to a similar level as the sham group (Fig. 2c, CLP 12 h + exo group 1.01 ± 0.1918 versus CLP 12 h group 0.6058 ± 0.07267, *p* = 0.0004), which may be a benefit from the undamaged mitochondria protected by huMSC-exos.

### Cardiomyocyte mitochondrial Ca^2+^ (_m_Ca^2+^) efflux was obstructed during sepsis and reversed by huMSCs-exo

**Fig. 3:**
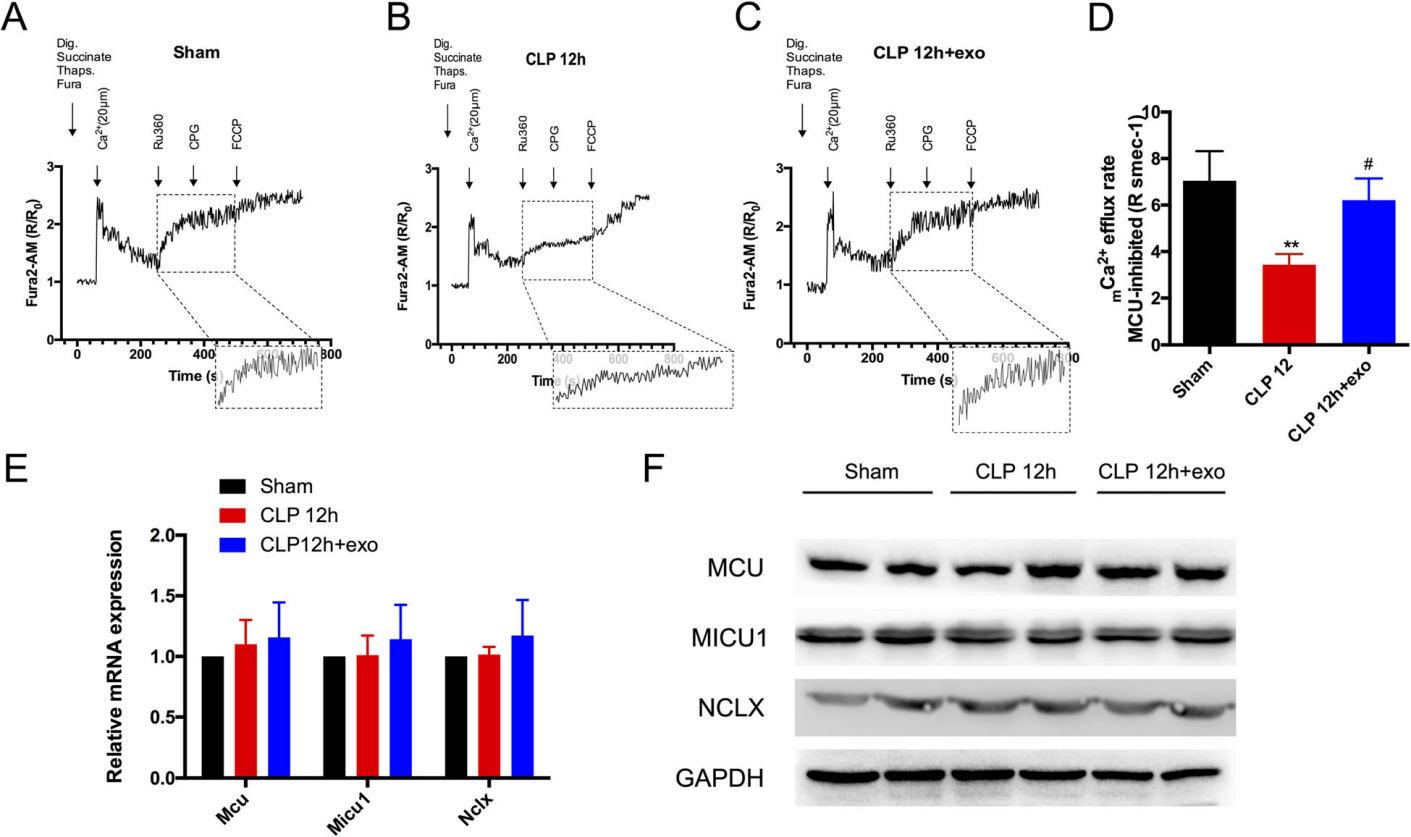
Assessment of cardiomyocyte mitochondrial Ca^2+^ (_m_Ca^2+^) uptake, efflux, and the corresponding regulator. **a**–**c**_m_Ca^2+^ uptake and efflux in isolated permeabilized ACMs; dig.: digitonin, thaps.: thapsigargin, Ru360: MCU inhibitor, CGP: NCLX inhibitor; the box shows a magnified _m_Ca^2+^ efflux tracing (260 s–360 s); R indicates the ratio of the ratiometric reporter Fura-2 (340/380 nm excitation and 510 nm emission). R/R_0_ indicates the ratio at each time point over the ratio at time 0. **d**_m_Ca^2+^ efflux rate, *n* = 3 replicates per group; **p < 0.01, #*p* < 0.05. **e** FQ-PCR detected the mRNA expression levels of *Mcu*, *Micu1*, and *Nclx* in the heart after CLP for 12 h. **f** Western blotting was used to detect MCU, MICU1, and NCLX expression in hearts 12 h after CLP. GAPDH was used as a loading control

To investigate how mitochondrial calcium overload of cardiomyocytes occurred in sepsis, we assessed the capacity of Ca^2+^ uptake and efflux in cardiomyocyte mitochondria. Adult cardiomyocytes (ACMs) isolated from sham-, CLP-, and huMSC-exo-treated mice were transferred to an intracellular-like medium, and 1 μM Fura2-AM was added to monitor extramitochondrial Ca^2+^. Thapsigargin was added to prevent SR and ER Ca^2+^ uptake. Therefore, the changes in the fluorescence intensity indicated Ca^2+^ influx or efflux into/out of the mitochondria. The results showed that after the Ca^2+^ bolus was added, the time for extramitochondrial Ca^2+^ to reach the low concentration occurred earlier in the CLP group than the sham group (Fig. 3a, b 60 s–260 s), which indicated that mitochondrial Ca^2+^ uptake in the CLP group was slightly faster than in the sham group. After MCU was inhibited with Ru360 at 260 s–360 s, Ca^2+^ uptake in the mitochondria was blocked, and the increase in extramitochondrial Ca^2+^ was representative of _m_Ca^2+^ efflux. These results showed that the peak concentration of extramitochondrial Ca^2+^ was much lower in the CLP group than the sham group during the same period of time, which indicates that the _m_Ca^2+^ efflux rate decreased significantly in the CLP group (Fig. 3a, b 260 s–360 s, d, CLP 12 h group 3.437 ± 0.4563 versus sham group 7.047 ± 1.274, *p* = 0.0083). In contrast, we found that although Ca^2+^ uptake by mitochondria in the huMSC-exo treatment group was not different from the CLP group (Fig. 3c 60 s–260 s), the _m_Ca^2+^ efflux rate increased to the level of the sham group (Fig. 3c 260 s–360 s, d, CLP 12 h + exo group 6.216 ± 0.9313 versus CLP 12 h group 3.437 ± 0.4563, *p* = 0.0268), which suggests that huMSC-exo maintained normal _m_Ca^2+^ efflux of cardiomyocyte mitochondria during sepsis.

Mitochondria regulate _m_Ca^2+^ distribution in two ways: cytoplasmic Ca^2+^ uptake is driven by ΔΨm and mediated primarily by MCU and MICU1 [27], and _m_Ca^2+^ efflux mediated by mitochondrial NCLX [28]. We assessed the mRNA expression of *Mcu*, *Micu1*, and *Nclx* 12 h after CLP and treatment with huMSCs-exo; the results showed there was little difference between these groups (Fig. 3e Mcu: sham group 1 (100%) versus CLP 12 h group 1.102 ± 0.2, *p* = 0.428 and CLP12h + exo group 1.157 ± 0.29 *p* = 0.686; Micu1: sham group 1 (100%) versus CLP 12 h group 1.01 ± 0.162, *p* = 0.917 and CLP12h + exo group 1.142 ± 0.28,*p* = 0.648; Nclx: sham group 1 (100%) versus CLP 12 h group 1.016 ± 0.064, *p* = 0.693 and CLP12h + exo group 1.172 ± 0.294, *p* = 0.545). The protein level of MCU, MICU1, and NCLX has also no difference between these groups (Fig. 3f, FigS1. B). These results indicate that the reason for abnormal _m_Ca^2+^ efflux 12 h after CLP may be suppression of NCLX activity.

### HuMSC-exo increased expression of PINK1 in cardiomyocytes during sepsis

**Fig. 4:**
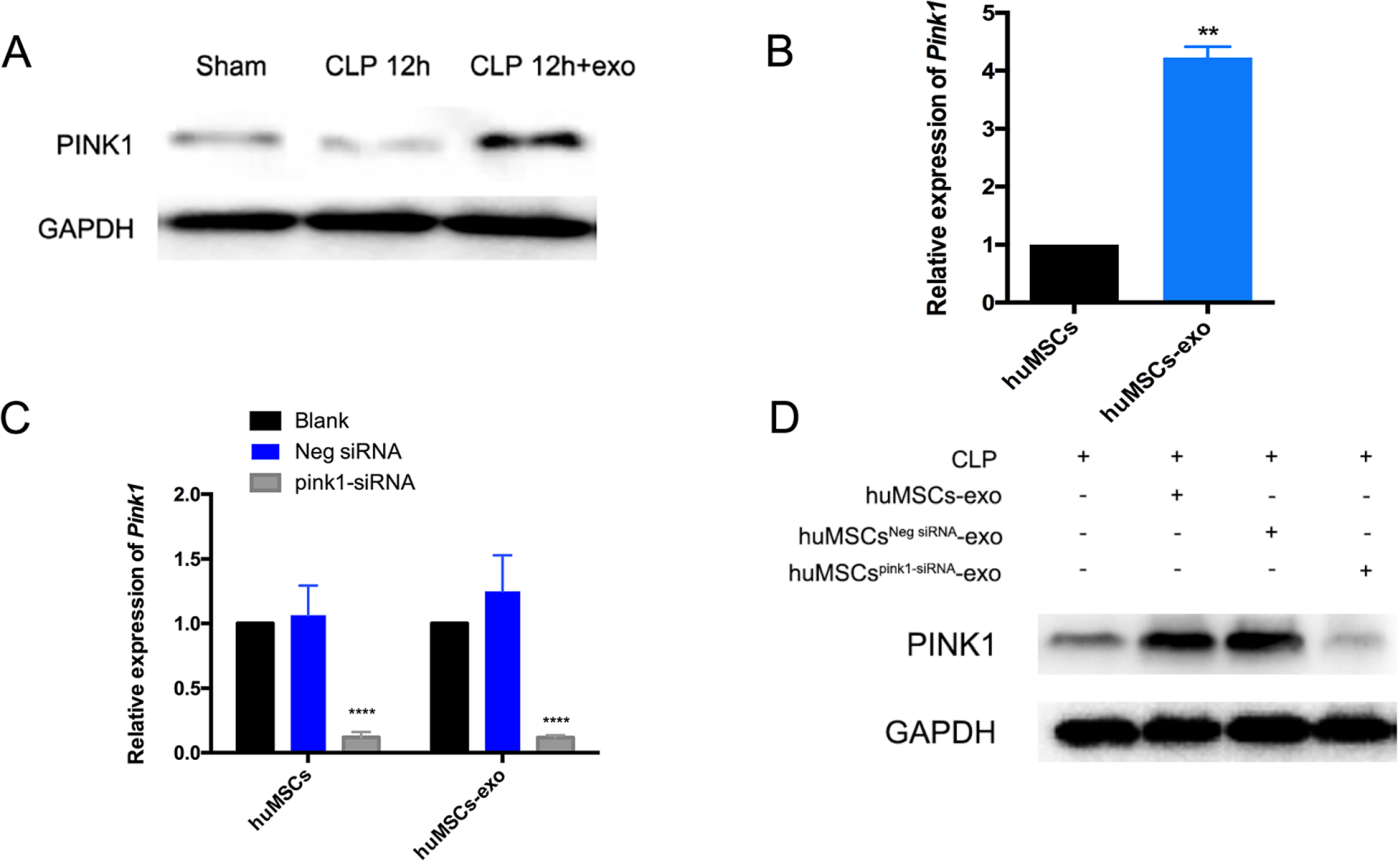
Sepsis decreased the expression of PINK1 in cardiomyocytes, and huMSC-exo transferred *Pink1* mRNA into cardiomyocytes to increase PINK1 expression. **a** Western blot detected PINK1 expression in hearts 12 h after CLP and after treatment with huMSC-exo; GAPDH was used as a loading control. **b**, **c** The relative expression of *Pink1* mRNA in huMSCs, huMSC-exo (**b**) and huMSCs, huMSC-exo transfected with siRNA (**c**); **p < 0.01 vs. huMSCs group, ****p < 0.0001 vs. Neg siRNA group. **d** Western blot analysis of PINK1 expression in the heart 12 h after CLP and after treatment with huMSC-exo or huMSC-exo transfected with siRNA; GAPDH was used as a loading control

Previous reports indicated that PINK1 regulated the activity of NCLX in neurons, but whether PINK1 also regulated the activity of NCLX in cardiomyocytes was not known. We found that expression of PINK1 decreased significantly 12 h after CLP (Fig. 4a), which is consistent with the abnormal _m_Ca^2+^ efflux results and suggests that the decrease in PINK1 expression in cardiomyocytes is associated with a reduction in NCLX activity. We also found that PINK1 expression increased in septic mouse cardiomyocytes after huMSC-exo treatment (Fig. 4a, FigS1. C), which further confirmed the relationship between PINK1 expression and NCLX activity. To further examine the source of increased PINK1 in cardiomyocytes after huMSC-exo treatment, we detected *Pink1* mRNA in huMSCs and their exosomes and found that huMSC-exo carried more *Pink1* mRNA than huMSCs (Fig. 4b, huMSC-exo group 4.225 ± 0.1875 versus huMSCs group 1 (100%), *p* = 0.0011).

We used *Pink1*-specific siRNA to inhibit *Pink1* expression in huMSCs to confirm whether huMSC-exo increased PINK1 expression in recipient cardiomyocytes by transferring *Pink1* mRNA from huMSCs. *Pink1* siRNA dramatically inhibited *Pink1* expression in huMSCs and their exosomes (Fig. 4c, huMSCs: *Pink1*-siRNA group 0.1187 ± 0.0421 versus Neg siRNA 1.064 ± 0.229, *p* < 0.0001; huMSC-exo: *Pink1*-siRNA group 0.117 ± 0.019 versus Neg siRNA 1.249 ± 0.281, *p* < 0.0001), and PINK1 expression in ACMs isolated from mice treated with *Pink1*-inhibited huMSC exosomes remained at a low level (Fig. 4d). These results suggest that the increase in PINK1 expression in recipient cardiomyocytes was due to the *Pink1* mRNA carried in huMSC-exo.

### HuMSC-exo with inhibited *Pink1* could not reverse sepsis-induced _m_Ca^2+^ efflux obstruction or mitochondrial damage

**Fig. 5:**
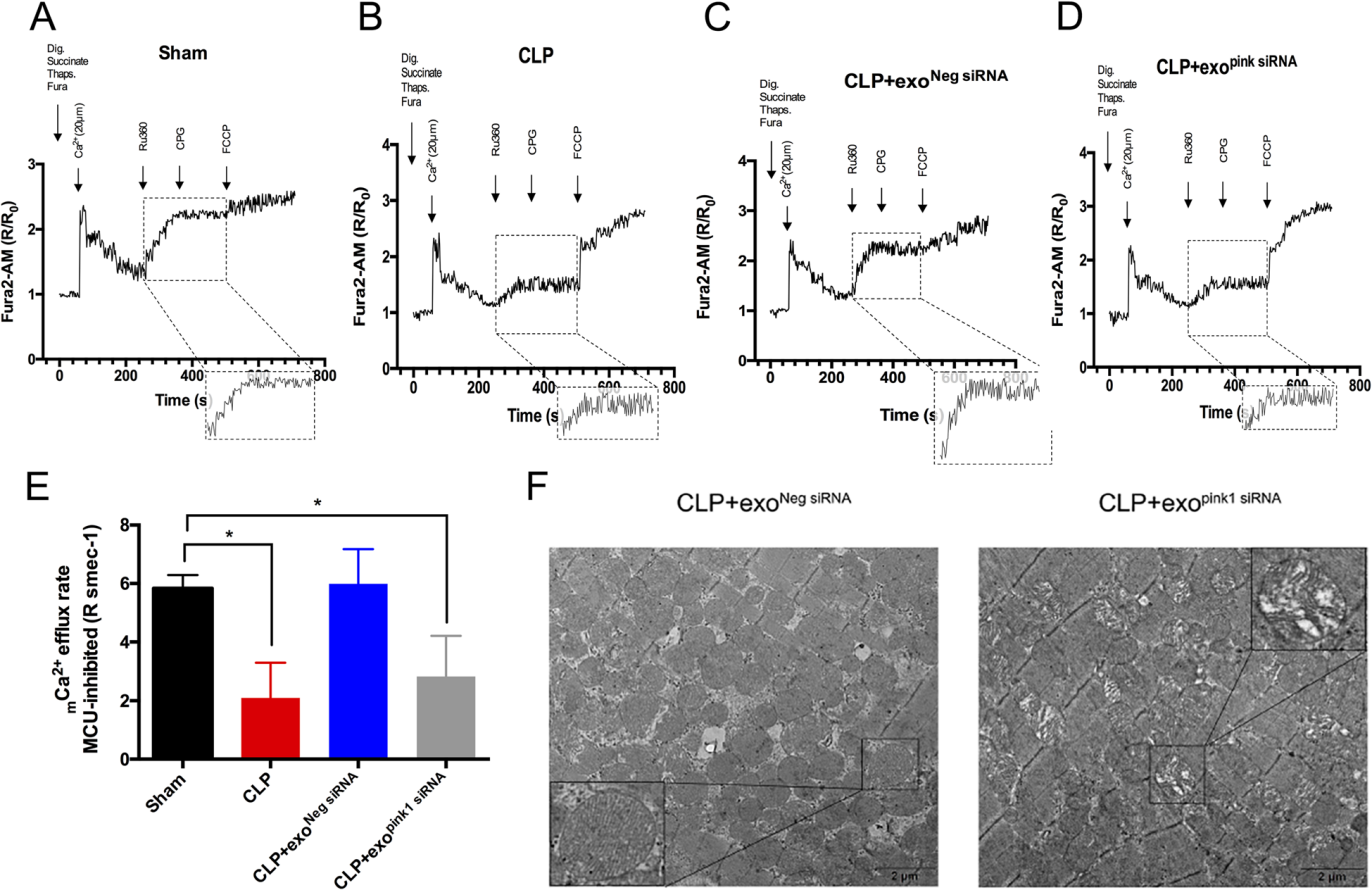
HuMSC-exo with inhibited PINK1 could not reverse sepsis-induced _m_Ca^2+^ efflux obstruction or mitochondrial damage. **a**, **d**_m_Ca^2+^ uptake and efflux in isolated permeabilized ACMs; dig.: digitonin, thaps.: thapsigargin, Ru360: MCU inhibitor, CGP: NCLX inhibitor; the box shows a magnified _m_Ca^2+^ efflux tracing (260 s–360 s); R indicates the ratio of the ratiometric reporter Fura-2 (340/380 nm excitation and 510 nm emission). R/R_0_ indicates the ratio at each time point over the ratio at time 0. **e**_m_Ca^2+^ efflux rate, *n* = 3 replicates per group, *p < 0.05. **f** Mitochondrial architecture observed with TEM after treatment with siRNA-transfected huMSC-exo, imaged at a × 10,000 magnification (scale bars, 2 μm), and the box in the image is a local magnification

To further validate the relationship of PINK1 to NCLX-mediated _m_Ca^2+^ efflux, we monitored the _m_Ca^2+^ efflux rate of ACMs from mice treated with *Pink1*-inhibited huMSC-exos. No significant difference was detected between the CLP + exo^pink1 siRNA^ and CLP groups, but these groups were decreased significantly compared to the sham group (Fig. 5a, b, d, e, CLP + exo^pink1 siRNA^ groups 2.829 ± 1.378 versus CLP group 2.098 ± 1.195, *p* = 0.8505 and sham group 5.836 ± 0.4564, *p* = 0.043). In contrast, the _m_Ca^2+^ efflux rate in the CLP treated with exo^Neg siRNA^ group was the same as the sham group (Fig. 5a, c, e, sham group 5.836 ± 0.4564 versus CLP+ exo^Neg siRNA^ group 5.988 ± 1.192, *p* = 0.998). These results suggest that the loss of *Pink1* mRNA attenuated the ability of huMSC-exos to recover _m_Ca^2+^ efflux. Whether this loss also weakened the ability of huMSC-exos to protect mitochondria from sepsis-induced damage was not clear. We observed mitochondrial morphology using TEM and found that the mitochondrial cristae number still decreased and the arrangement was disordered in CLP mice with exo^pink1 siRNA^ treatment (Fig. 5f). This result indicates that the mitochondrial protection of huMSC-exos was greatly weakened after *Pink1* mRNA inhibition.

### PINK1 may regulate NCLX-mediated _m_Ca^2+^ efflux by affecting PKA activity after transfer from huMSC-exo to cardiomyocytes

**Fig. 6:**
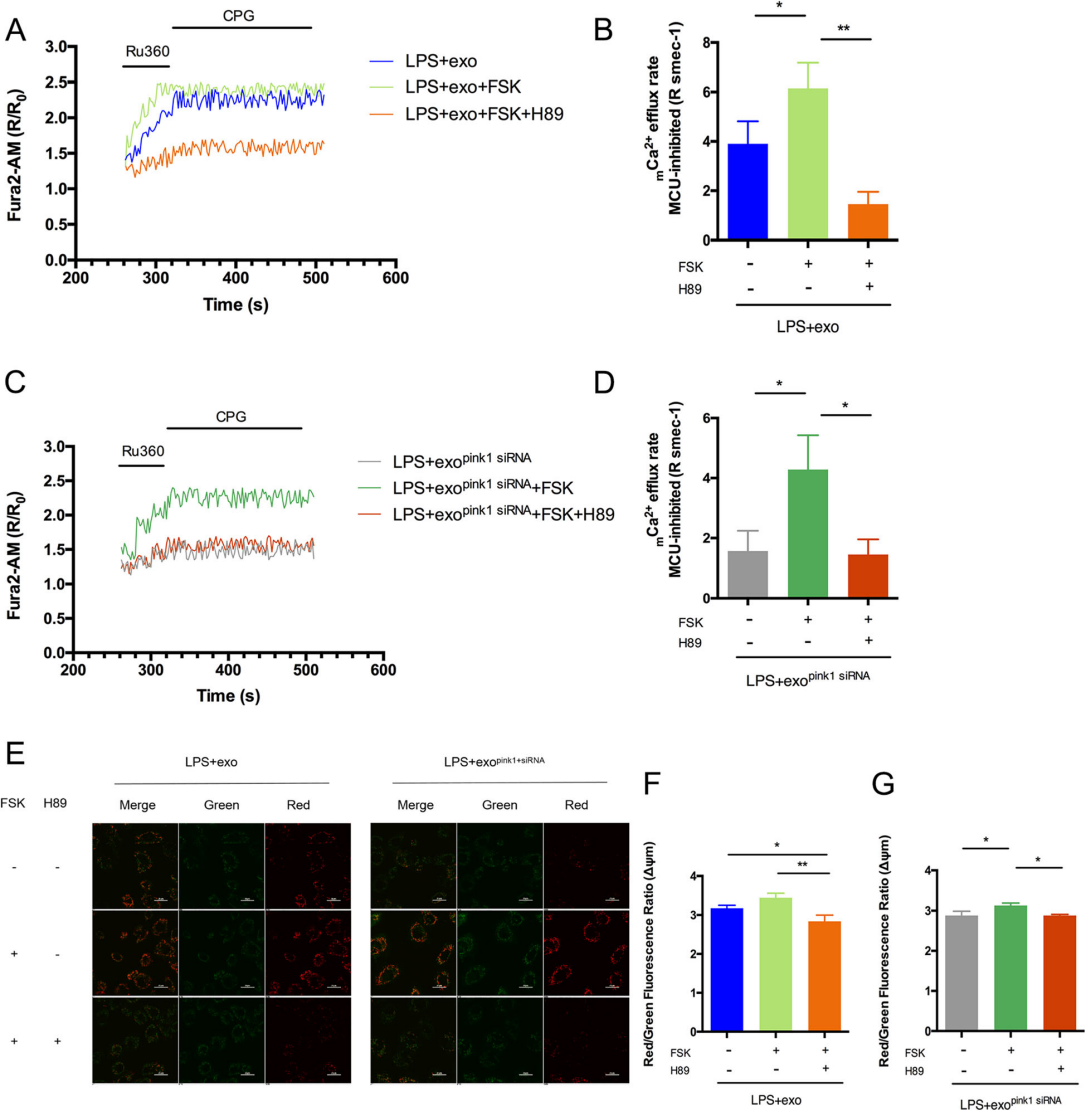
PINK1 transferred from huMSC-exo regulated _m_Ca^2+^ efflux by affecting PKA activity in cardiomyocytes. **a** AC16 cells were treated with LPS and huMSC-exo, and FSK or FSK and H89 were added. The _m_Ca^2+^ efflux of cells was detected; Ru360: MCU inhibitor, CGP: NCLX inhibitor. **b** Average _m_Ca^2+^ efflux rates of Fig. 6a, *n* = 3 replicates per group; *p < 0.05, **p < 0.01. **c** AC16 cells were treated with LPS and *Pink1*-inhibited huMSC-exo, after which FSK or FSK and H89 were added. The _m_Ca^2+^ efflux of cells was assessed; Ru360: MCU inhibitor, CGP: NCLX inhibitor. **d** Average _m_Ca^2+^ efflux rates of Fig. 6c, n = 3 replicates per group; *p < 0.05. **e**–**g** Mitochondrial membrane potential analysis was measured using a fluorescence probe JC-1 assay system. The images were captured using confocal microscopy, Red: mitochondrial J–aggregates, green: mitochondrial monomers (**e**), and the ratio of red/green fluorescence represented the level of Δψm (**f**, **g**). A high ratio indicates a high mitochondrial membrane potential; *p < 0.05, **p < 0.01

The above results confirmed the effect of PINK1 on NCLX-mediated _m_Ca^2+^ efflux, but no evidence showed a direct interaction between PINK1 and NCLX. In contrast, many studies argued against a direct interaction of PINK1 and NCLX [13, 29]. One study showed that PINK1-deficient cells exhibited PKA inhibition and NCLX-mediated _m_Ca^2+^ efflux impairment, which were fully rescued by activated PKA [13]. We also found that the PKA activity significantly reduced at CLP 12 h (FigS3. A). To determine whether PINK1 regulated NCLX-mediated _m_Ca^2+^ efflux via PKA, we used forskolin (FSK) and H89 to activate and inhibit PKA, respectively. The result showed that FSK could increase PKA activity significantly, and H89 could suppress PKA activity greatly (FigS3. B, C). AC16 cells were treated with LPS and huMSC-exo, and the _m_Ca^2+^ efflux rate was monitored. The results showed that PKA activation by FSK (Fig. 6a, b light green) enhanced the _m_Ca^2+^ efflux rate compared to treatment with huMSC-exos alone (Fig. 6a, b blue, LPS + exo + FSK group 6.15 ± 1.03 versus LPS + exo group 3.901 ± 0.918, *p* = 0.0448), but the _m_Ca^2+^ efflux rate decreased significantly when FSK and H89 were co-applied (Fig. 6a, b light orange, LPS + exo + FSK + H89 group 1.578 ± 0.613 versus LPS + exo + FSK group 6.15 ± 1.03, *p* = 0.0016). Consistent with a previous study [13], we found that FSK greatly recovered the decrease in _m_Ca^2+^ efflux rate caused by the absence of PINK1, but H89 abolished the effect of FSK (Fig. 6c, d, LPS + exo^pink1 siRNA^ + FSK group 4.283 ± 1.145 versus LPS + exo^pink1 siRNA^ group 1.568 ± 0.676, *p* = 0.0158, and LPS + exo^pink1 siRNA^ + FSK + H89 group 1.454 ± 0.503, *p* = 0.0131). The ΔΨm also increased after FSK treatment, and this effect was eliminated by H89 (Fig. 6e, f, g, LPS + exo^pink1 siRNA^ + FSK group 3.13 ± 0.06 versus LPS + exo^pink1 siRNA^ group 2.882 ± 0.108, *p* = 0.0138, and LPS + exo^pink1 siRNA^ + FSK + H89 group 2.883 ± 0.025, *p* = 0.0141). These results suggest that PKA is essential for PINK1 regulation of NCLX-mediated _m_Ca^2+^ efflux.

## Discussion

The heart is an important target organ in severe sepsis/septic shock, and its dysfunction manifests in many different ways in sepsis, including left and/or right ventricular impairment during systole or diastole, inadequate cardiac output and oxygen delivery, or primary myocardial cellular injury [30]. Sepsis with cardiac dysfunction increases mortality by 50% [31], which should arouse our attention. Fluid resuscitation is used clinically to improve cardiac perfusion, cardiac output, and systolic function at the early stage of sepsis to support the heart, but there is no specific treatment. This lack of strategy is likely due to the unclear and complicated mechanism of sepsis-induced cardiac dysfunction, which also explains the lack of drugs aimed at corresponding targets. Previous studies indicated that the main proposed mechanisms underlying the pathophysiology of myocardial dysfunction in sepsis support a prominent role of functional, rather than anatomical, abnormalities [31]. The clinically significant pathophysiological changes in cardiac function that occur early during sepsis may occur in the absence of cellular hypoxia or histological changes, which suggests that severe metabolic derangement underlies the development of septic cardiomyopathy [32–35]. This opinion is consistent with the results of the present study, which showed cardiomyocyte mitochondrial damage 12 h after CLP, which is much earlier than cardiomyocyte apoptosis (24 h after CLP). This early damage explains our use of 12 h after CLP as the investigational time point. Mitochondrial damage is an important trigger that causes apoptosis, which is characterized by a change in mitochondrial architecture (swelling, internal vesicle formation, and abnormalities in cristae), mitochondrial DNA damage, and elevation in mitochondrial permeability transition [9, 36, 37]. Mitochondria are the power houses of cells that provide continuous energy for heart activity, and injury inevitably induces metabolic derangement and a decrease in ATP production, which weakens myocardial contractility and eventually causes heart dysfunction.

The reported mechanisms of sepsis-induced cardiomyocyte mitochondrial dysfunction include energy metabolism disorder [38], calcium overload [39], autophagy [40], and mitochondrial inner membrane damage [41]. Calcium acts as an important intracellular second messenger and plays a key role in cardiac contractility. Mitochondria are one of the largest calcium pools in cardiomyocytes and alleviate the high concentration of Ca^2+^ via the uptake of intracellular Ca^2+^ (_i_Ca^2+^) into the mitochondrial matrix. However, when _m_Ca^2+^ exceeds full capacity, the mitochondrial permeability transition pore (MPTP) irreversibly open, and the ΔΨm is reduced, which induces mitochondrial dysfunction [39]. Normal mitochondria need ΔΨm to maintain their function, and a decreased ΔΨm is an important cause of apoptosis. Calcium overload is an upstream event of mitochondrial depolarization [13]. Therefore, mitochondrial calcium overload may be an important mechanism of mitochondrial dysfunction in septic hearts. This hypothesis was further confirmed in the present study, which found cardiomyocyte impairment of _m_Ca^2+^ shuttling 12 h after CLP, much earlier than apoptosis occurred.

There are two processes that cause mitochondrial calcium overload: excessive calcium uptake into mitochondria, which is mainly driven by MCU; or a decrease in calcium efflux from the mitochondrial matrix to the cytoplasm [27, 28]. NCLX is the most important regulator of mitochondrial calcium efflux and may be the primary mechanism for _m_Ca^2+^ extrusion in excitable cells [10]. Abnormal MCU or NCLX causes _m_Ca^2+^ shuttling system disorder, but research showed that an MCU knockout yielded a relatively mild phenotype, and a conditional knockout of NCLX led to rapid fatal heart failure [28], which suggests that NCLX-mediated efflux is necessary to maintain homeostatic _m_Ca^2+^ levels in cardiomyocytes and for survival. We found no change in Ca^2+^ uptake by cardiomyocyte mitochondria in the early stage of sepsis, but _m_Ca^2+^ efflux was obviously abnormal. This result suggests that sepsis-induced cardiac dysfunction is caused by mitochondrial calcium overload resulting from reduced mitochondrial calcium efflux. However, no difference in NCLX expression was found between the CLP and sham group mice in our study, which suggests that the reason for dysfunction of mCa^2+^ efflux after CLP was related to the suppressed NCLX activity.

The present study found that expression of PINK1, a key protein associated with NCLX-mediated _m_Ca^2+^ efflux, decreased significantly in CLP mouse hearts. PINK1 is a serine/threonine kinase that was initially linked to the pathogenesis of a familial form of Parkinson’s disease [42, 43], and its loss-of-function mutations cause dopaminergic neuron mitochondrial calcium overload, which makes cells particularly vulnerable to injury [14]. PINK1 KO mice developed left ventricular dysfunction and cardiac hypertrophy, which led to pressure overload-induced heart failure. Mitochondria from PINK1 KO hearts exhibited an altered morphology, reduced mitochondrial membrane potential, and decreased oxidative phosphorylation, which induced oxidative stress and increased cardiomyocyte apoptosis [42, 44, 45]. Although PINK1 is associated with NCLX-mediated _m_Ca^2+^ efflux, the interaction between PINK1 and NCLX is indirect [29]. Previous studies demonstrated that the loss of PINK1 led to PKA inhibition and modulation of the NCLX phosphorylation site by PKA activation was essential for NCLX activity [13, 46]. Our results also showed that sepsis decreased PKA activity. Therefore, we hypothesized that the PINK1-PKA-NCLX axis in cardiomyocytes regulates _m_Ca^2+^ efflux, similar to neurons, and abnormalities in this axis cause sepsis-induced mitochondrial calcium overload in cardiomyocytes.

Next, we explored the role of huMSCs-exo in heart protection during sepsis and whether PINK1-PKA-NCLX axis may be a therapeutic target. Firstly, we confirmed that exosomes secreted from huMSCs attenuated sepsis-induced cardiomyocyte injury via mitochondrial repair. A more regular mitochondria morphology and greater ATP production means a more integrated structure and more powerful function. Secondly, our results showed that huMSC-exo reversed the sepsis-induced reduction in cardiomyocyte _m_Ca^2+^ efflux. Notably, we found the huMSC-exo carried more *Pink1* mRNA than the huMSCs themselves, and this mRNA was transferred from huMSC-exo to recipient cardiomyocytes to increase PINK1 expression. PKA activity was also increased after treatment with huMSC-exo. When *Pink1* mRNA contained in huMSC-exo was inhibited, huMSC-exo lost the ability of _m_Ca^2+^ efflux regulation, and the PKA activity of recipient cardiomyocytes was not change in sepsis mouse hearts. Activated PKA rescued the LPS-induced reduction in _m_Ca^2+^ efflux and ΔΨm when huMSC-exo lost their ability to regulate _m_Ca^2+^ efflux, and this effect abolished after PKA activity inhibition by H89. These results suggest that huMSC-exos ameliorate sepsis-induced cardiomyocyte mitochondrial calcium overload via the transfer of *Pink1* mRNA into cardiomyocytes to restore PINK1 expression, and upregulated PINK1 increases PKA activity to activate NCLX-mediated _m_Ca^2+^ efflux and protect cardiomyocytes from injury.

Research on PINK1 revealed that its multiple functions extend well beyond mitochondrial calcium efflux regulation. PINK1 is a crucial player in the mitochondrial quality control pathway [42, 47], and it promotes damaged mitochondria elimination via Parkin-dependent [48, 49] or Parkin-independent [42, 50] mitophagy. Therefore, in our research, after huMSCs-exo increase PINK1 expression in septic cardiomyocytes, is it possible that huMSCs-exo eliminate the damaged mitochondria caused by mitochondrial calcium overload via activation of PINK1-related mitophagy to protect cardiomyocytes? This hypothesis was not investigated in this study, but it provides direction for further research.

In conclusion, we first confirmed that sepsis-induced PINK1-PKA-NCLX axis abnormalities in cardiomyocytes are the main cause of cardiomyocyte mitochondrial calcium overload and heart dysfunction. We also found that exosomes secreted from huMSCs transferred *Pink1* mRNA to cardiomyocytes and rescued the decrease in _m_Ca^2+^ efflux-induced mitochondrial calcium overload by restoring the PINK1-PKA-NCLX axis. One important limitation of this study is that we did not examine whether PINK1-dependent mitophagy is involved in the cardioprotective effects of huMSC-exo. Nevertheless, our data support PINK1 as a therapeutic target to protect cardiomyocyte mitochondria, and the application of huMSC-exo is a promising strategy to combat sepsis-induced heart dysfunction.

## Supplementary Information

**Additional file 1: Table S1.** Index of Left Ventricular Function measured by Echocardiography. **Supplementary Figure 1.** Quantitative analysis of protein expression. A: The exosome markers, *p < 0.05, ***p < 0.001 compared with 3 μg group; B: The expression of MCU, MICU1 and NCLX at 12 h after CLP and treatment with huMSCs-exo; C: The PINK1 expression at 12 h after CLP and treatment with huMSCs-exo, **p < 0.01 compared with sham group, ####*p* < 0.0001 compared with CLP 12 h group; D: The PINK1 expression at 12 h after CLP and treatment with huMSCs-exo or *Pink1* deficient huMSCs-exo, ****p < 0.0001. **Supplementary Figure 2.** The PKH26-labeled exosomes were absorbed by cardiomyocytes (magnification: 400x). Red: PKH26, Green: cTnI, Blue: DAPI. **Supplementary Figure 3.** Detection of PKA activity. A: The PKA activity at 12 h after CLP and treatment with huMSCs-exo; B, C. The PKA activity after treated with FSK and H89.

## Data Availability

The data that support the findings of this study are available from the corresponding author upon reasonable request.

## Abbreviations

huMSCs: Human umbilical cord mesenchymal stem cells
CLP: Cecal ligation and puncture
TEM: Transmission electron microscope
huMSCs-exo: Exosomes isolated from huMSCs
NCLX: Mitochondrial Na^+^/Ca^2+^ exchanger
PINK1: PTEN-induced putative kinase 1
HBDH: α-Hydroxybutyrate dehydrogenase
CK: Creatine kinase
cTnI: Cardiac troponin-I
FBS: Fetal bovine serum
PKA: Protein kinase A
EF: Ejection fraction
ACMs: Adult cardiomyocytes
SLP-2: Stomatin-like protein 2
PKC: Protein kinase C
