# Supplementary material for: PINK1 contained in huMSC-derived exosomes prevents cardiomyocyte mitochondrial calcium overload in sepsis via recovery of mitochondrial Ca2+ efflux
Source: Stem Cell Res Ther. 2021 May 6;12:269. doi: 10.1186/s13287-021-02325-6 (PMC8101124; doi:10.1186/s13287-021-02325-6)
Supplement: Supplementary file 1 — Additional file 1: Table S1. Index of Left Ventricular Function measured by Echocardiography. Supplementary Figure 1. Quantitative analysis of protein expression. A: The exosome markers, *p < 0.05, ***p < 0.001 compared with 3 μg group; B: The expression of MCU, MICU1 and NCLX at 12 h after CLP and treatment with huMSCs-exo; C: The PINK1 expression at 12 h after CLP and treatment with huMSCs-exo, **p < 0.01 compared with sham group, ####p < 0.0001 compared with CLP 12 h group; D: The PINK1 expression at 12 h after CLP and treatment with huMSCs-exo or Pink1 deficient huMSCs-exo, ****p < 0.0001. Supplementary Figure 2. The PKH26-labeled exosomes were absorbed by cardiomyocytes (magnification: 400x). Red: PKH26, Green: cTnI, Blue: DAPI. Supplementary Figure 3. Detection of PKA activity. A: The PKA activity at 12 h after CLP and treatment with huMSCs-exo; B, C. The PKA activity after treated with FSK and H89. [file 13287_2021_2325_MOESM1_ESM.docx]

Table S1. Index of Left Ventricular Function measured by Echocardiography

|  | LVIDd (cm) | LVIDs (cm) | EDV (μl) | ESV (μl) | SV (μl) |
| --- | --- | --- | --- | --- | --- |
| Sham | 0.3433±0.03212 | 0.1447±0.0071 | 104.9±29.09 | 8.366±1.188 | 96.51±30.13 |
| CLP 12h | 0.28±0.01732 | 0.18±0.0052 | 57.74±10.62 | 15.85±1.317 | 41.89±10.03 |
| CLP 12h+exo | 0.3333±0.0252 | 0.16±0.005 | 95.81±20.93 | 11.22±1.029 | 84.59±21.58 |

LVIDd: Left ventricular internal dimension-diastole; LVIDs: Left ventricular internal dimension in systole; EDV: End-Diastolic Volume; ESV: End-systole Volume; SV: stroke volume


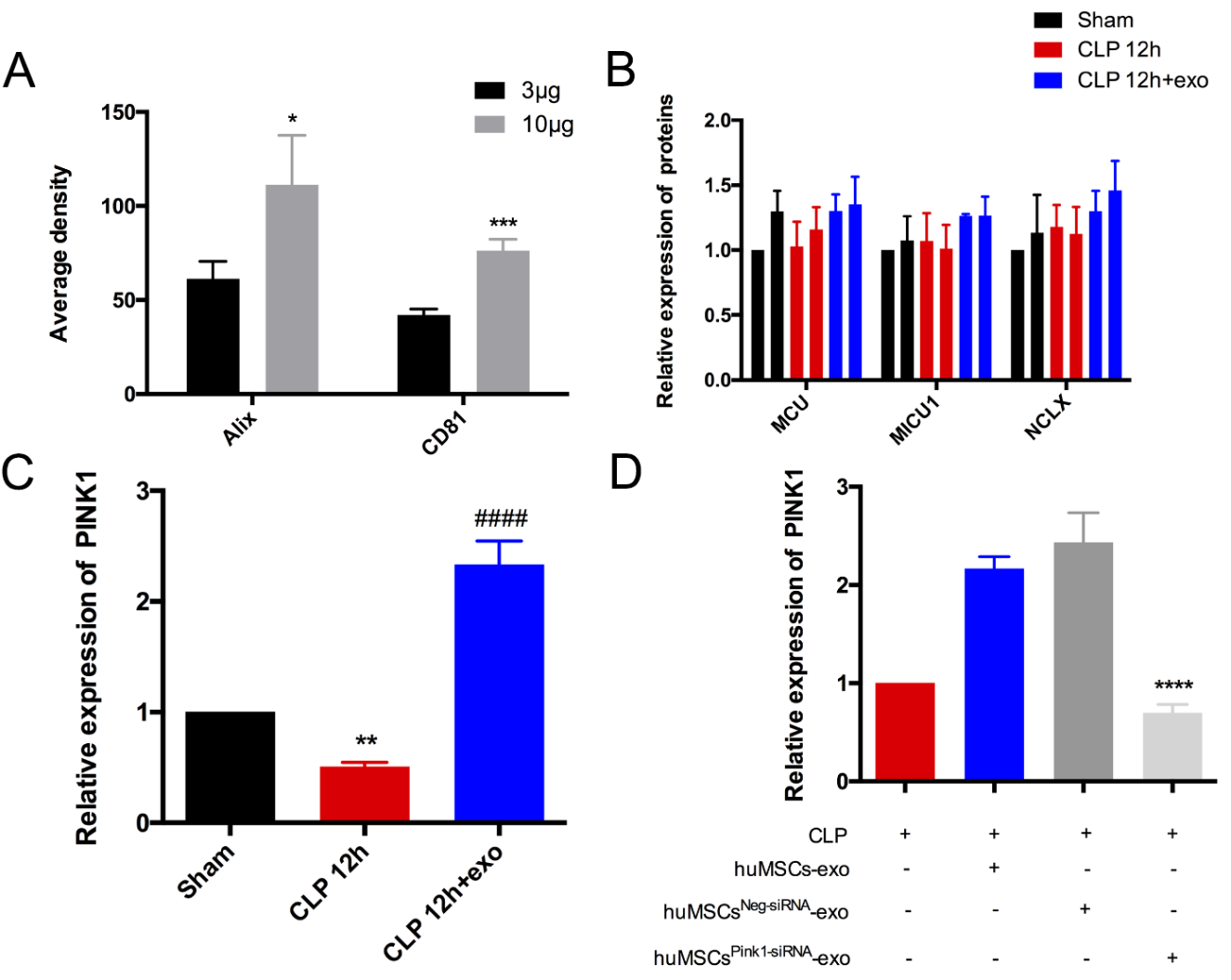


Supplementary Figure 1 Quantitative analysis of protein expression. A: The exosome markers, **p*<0.05, ****p*<0.001 compared with 3μg group; B: The expression of MCU, MICU1 and NCLX at 12hr after CLP and treatment with huMSCs-exo; C: The PINK1 expression at 12hr after CLP and treatment with huMSCs-exo, ***p*<0.01 compared with Sham group, ####*p*<0.0001 compared with CLP 12h group; D: The PINK1 expression at 12hr after CLP and treatment with huMSCs-exo or *Pink1* deficient huMSCs-exo, *****p*<0.0001.


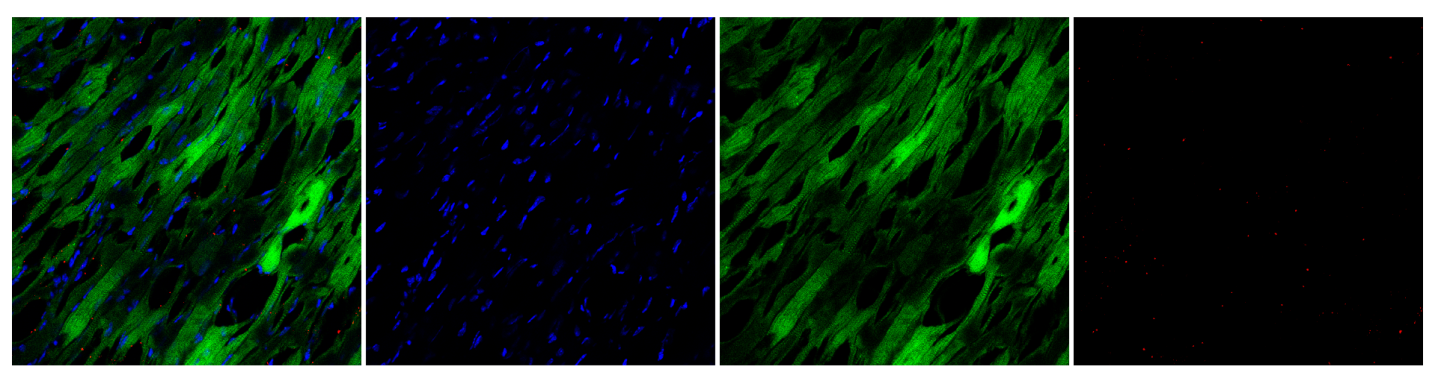


Supplementary Figure 2 The PKH26-labeled exosomes were absorbed by cardiomyocytes (magnification: 400x). Red: PKH26, Green: cTnI, Blue: DAPI


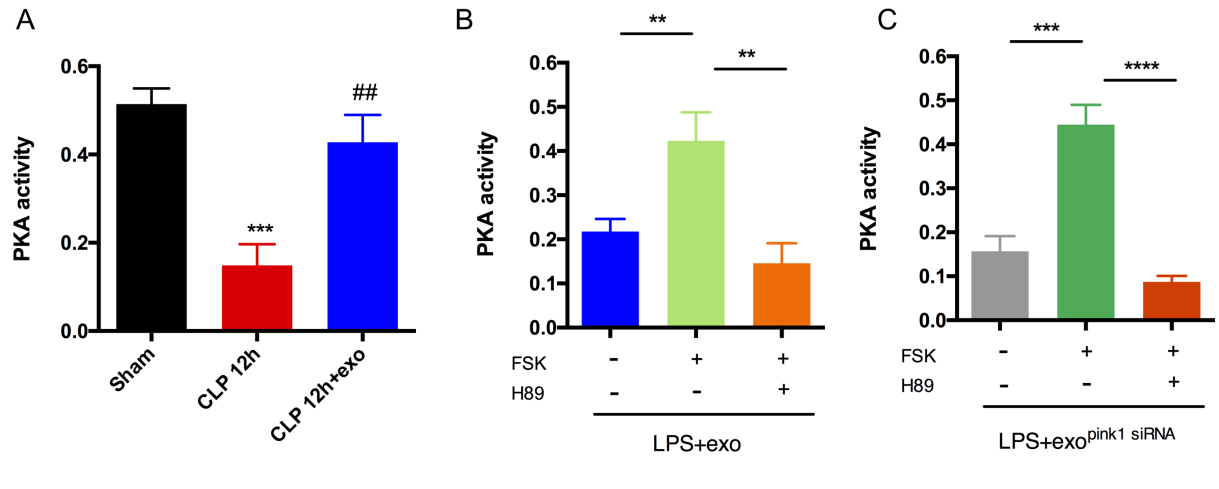


Supplementary Figure 3 Detection of PKA activity. A: The PKA activity at 12hr after CLP and treatment with huMSCs-exo; B, C. The PKA activity after treated with FSK and H89.
